# A Monte Carlo-Based Framework for Two-Stage Stochastic Programming: Application to Bond Portfolio Optimization

**DOI:** 10.3390/e27111118

**Published:** 2025-10-30

**Authors:** Hissah Albaqami, Mehdi Mrad, Anis Gharbi, Munevver Mine Subasi

**Affiliations:** 1 Department of Mathematics and Systems Engineering, Florida Institute of Technology, Melbourne, FL 32901, USA; h.hassh@tu.edu.sa; 2 Department of Mathematics, College of Science, Turabah University College, Taif University, Turbah 29731-9086, Saudi Arabia; 3 Essect School of Business, University of Tunis, Tunis 1089, Tunisia; mehdi.mrad@essect.u-tunis.tn; 4 Business Analytics and Decision Making Lab, Tunis Business School, University of Tunis, Bir El Kassaa 2059, Tunisia; 5 Department of Industrial Engineering, College of Engineering, King Saud University, Riyadh 11421, Saudi Arabia; a.gharbi@ksu.edu.sa

**Keywords:** stochastic programming, bond portfolio optimization, Monte Carlo simulation, sample average approximation (SAA), mixed-integer linear programming (MILP), decision-making under uncertainty, entropy, information theory, uncertainty quantification

## Abstract

This paper presents a Monte Carlo simulation-based approach for solving stochastic two-stage bond portfolio optimization problems. The main objective is to optimize the cost of the bond portfolio while making decisions on bond purchases, holdings, and sales under random market conditions such as interest rate fluctuations and liabilities. The proposed algorithm identifies the number of randomly generated scenarios required to convert the stochastic problem into a deterministic one, subsequently solving it as a Mixed-Integer Linear Program. The practical relevance of this research is shown through an application of the proposed method to a real-world bond market. The results indicate that the proposed approach successfully minimizes costs and meets liabilities, providing a robust solution for bond portfolio optimization.

## 1. Introduction

This paper focuses on the use of two-stage stochastic programming in bond portfolio optimization. In the realm of financial decision-making, uncertainty plays a critical role, particularly in bond portfolio optimization where market parameters such as interest rates and liabilities are inherently volatile. Two-stage stochastic programming provides a powerful modeling framework for bond portfolio optimization problems by enabling early-stage decisions followed by adaptive responses to realized outcomes. Bond portfolios, even those constructed conservatively, are subject to substantial risks due to fluctuating interest rates, bond prices, cash requirements, liabilities, and potential defaults. Effective management of these portfolios requires robust optimization techniques capable of handling such uncertainties.

The portfolio selection problem is one of the most prominent research topics in modern finance (Ardakani 2024 [1]; Yan et al., 2023 [2]; Novais et al., 2022 [3]). Finding a combination of assets that satisfies an investor’s needs is the main goal of portfolio selection problem theory. The theory of portfolio optimization was developed by Markowitz (1952) [4]. Bond portfolios are subject to major uncertainties and risks even when they are conservatively assembled. Some of the uncertainties associated with the bond portfolio model can be seen as uncertainties in the interest rates and their effects on bond prices, including the cash requirements or liabilities of the portfolio and the possibility of bond re-calls or defaults due to bankruptcy, to name just a few (Shapiro 1988 [5]). The dynamic model of the bond portfolio problem proposed by Bradley and Crane (1972) [6] can be viewed as a multi-stage decision problem in which actions such as “buying, holding, or selling” bonds are taken at successive time points.

In this paper, we propose a Monte Carlo simulation-based methodology to solve a two-stage stochastic bond portfolio optimization problem, incorporating concepts from uncertainty quantification to guide scenario generation and improve decision quality. Specifically, we use the sample average approximation (SAA) approach to reduce the uncertainty space by generating a representative set of market scenarios (Hu et al. 2020 [7]; Jiang and Li 2021 [8]). SAA leverages iterative random sampling from input distributions to estimate objective function values, thereby directing the optimization process (Bertsimas et al., 2018 [9]). Through the systematic generation of random scenarios, the methodology transforms the stochastic framework into a deterministic problem structure, enabling resolution via Mixed-Integer Linear Programming (MILP) techniques implemented through commercial optimization software. An entropy-based approach can also be used for portfolio optimization (Novais et al., 2022 [3]; Mercurio et al., 2020 [10]).

Let *x* and *y* designate the first- and second-stage decision vectors, respectively, and let ξ be the random vector to be observed. Then, the decision-observation scheme (Prékopa 1995 [11]) is


“Decision on *x* → Observation of *ξ* → Decision on *y*.”

The second-stage problem is formulated under the assumption that the values of *x* and ξ are known:

(1) $$\begin{array}{cc} \text{min}\; & q^{T}y\\ s.t\\ \; & \;Tx+Wy=\xi \\ \; & \;y\geq 0 \end{array}$$

Assume that the first-stage decision vector *x* satisfies the deterministic constraints: Ax=b,x≥0, and let *F* be the set of all those *x* vectors for which problem (1) has a feasible solution for every possible value of the random vector ξ. Matrices $T\in R^{m_{2}\times n_{1}}$ associated with vector *x* and $W\in R^{m_{2}\times n_{2}}$ associated with vector *y* are called the technology matrix and the recourse matrix, respectively.

Let q(x,ξ) designate the optimum value of problem (1), and Q(x)=E[q(x,ξ)] be the recourse function. Then, the first-stage problem can be formulated as:

(2) $$\begin{array}{cc} \text{min}\; & c^{T}x+Q\left(x\right)\\ \text{subject to}\\ \; & \;Ax=b\\ \; & \;x\geq 0\\ \; & \;x\in F \end{array}$$

equivalently,

(3) $$\begin{array}{cc} \text{min}\; & c^{T}x+E\left[q\left(x,\;\xi \right)\right]\\ \text{subject to}\\ \; & \;Ax=b\\ \; & \;x\geq 0\\ \; & \;x\in F \end{array}$$

 where

$x\in R^{n_{1}}$: first-stage decision vector,
$y\in R^{n_{2}}$: second-stage decision vector,
$\xi \in R^{m_{2}}$: a random vector to be observed,
*F*: set of all $x\in R^{n_{1}}$ for which problem (1) has feasible solutions for every possible value of the random vector ξ,
$c\in R^{n_{1}}$: cost vector corresponding to the first-stage decision vector *x*,
$q\in R^{n_{2}}$: cost vector corresponding to the second-stage decision vector *y*,
$A\in R^{m_{1}\times n_{1}}$: coefficient matrix, and $b\in R^{m_{1}}$: right-hand side vector of the equality constraints.

Let x∈F and Ξ denote the support of the random vector ξ. Then, problem (3) has a finite optimum if, and only if, there exists a vector $z\in R^{m_{2}}$ such that $W^{T}z\leq q$. This assertion follows from the duality theory in linear programming (Prékopa 1995 [11]). If such vector *z* exists (*z* is dual feasible) and the expected value of the random variable ξ exists, then for every x∈F, the expectation E[q(x,ξ)] exists and is a convex function on the convex polyhedron *F* (Prékopa 1995 [11]).

One approach to solve problem (3) is to approximate E[q(x,ξ)] using the Monte Carlo simulation-based method (Shapiro 2001 [12]). Monte Carlo sampling constitutes a widely implemented methodology for addressing stochastic optimization challenges through iterative random sampling procedures. The approach estimates objective function values via repeated sampling from specified input distributions, utilizing these estimates to direct the optimization trajectory. This computational framework operates by generating substantial quantities of random samples from appropriate probability distributions—whether uniform, normal, exponential, or others—contingent upon problem characteristics. Once the samples have been generated, an appropriate optimization algorithm can be used to estimate the objective function and search for an optimal solution (Brandimarte 2014 [13]).

The motivation of this research stems from challenges in bond portfolio optimization under uncertainty, particularly in the Saudi Sukuk (bond) market. To address these challenges, we adopt and extend the two-stage stochastic programming framework by introducing a novel adaptive sampling rule, thereby combining methodological innovation with practical relevance. While two-stage stochastic programming combined with Monte Carlo simulation and sample average approximation (SAA) is well established in the literature, the originality of our study lies in extending these methods both methodologically and in application. Methodologically, we develop a novel adaptive sampling procedure that introduces a dual-condition stopping criterion based simultaneously on hypothesis tests of the sample mean and the sample variance across successive iterations. If both tests confirm equality, the algorithm terminates since enlarging the sample would not yield further improvement; otherwise, the sample size is expanded and the procedure is repeated. This ensures that the sample size is increased only when statistically significant improvements are observed, thereby enhancing computational efficiency without compromising solution quality. To the best of our knowledge, such a dual statistical stopping mechanism has not been applied before in stochastic programming. From an application perspective, although the SAA approach is common in stochastic programming in general, its direct use in bond portfolio optimization remains virtually unexplored. Existing works on bond portfolios under uncertainty (Alreshidi et al., 2020 [14]; Alkailany et al., 2022 [15]) typically rely on fixed scenario trees or deterministic recourse models. By adopting the SAA in this context, and further innovating with the proposed dual statistical stopping criterion, our paper extends a general methodology into a new and underdeveloped application area, thereby contributing both methodological novelty and practical relevance.

The remainder of this paper is organized as follows: Section 2 reviews the relevant literature on bond portfolio optimization and stochastic programming. Section 3 presents the basic mathematical models for the studied problem. Section 4 details the methodology of the Monte Carlo simulation approach. Section 5 presents a case study applying the proposed method to the Saudi Sukuk market and discusses its results and implications. Finally, Section 6 concludes the paper with a summary of findings and potential directions for future research.

## 2. Literature Review

The portfolio selection problem has been a crucial research topic in modern finance, with the primary goal of identifying asset combinations that fulfill investor needs. The foundational theory of portfolio optimization was introduced by Markowitz (1952) [4], establishing a basis for understanding and managing the uncertainties and risks inherent in bond portfolios. These risks include fluctuating interest rates, bond prices, cash requirements, liabilities, and the potential for bond recalls or defaults (Shapiro 1988 [5]).

Bradley and Crane (1972) [6] pioneered a dynamic bond portfolio model, conceptualizing it as a multi-stage decision process involving sequential actions such as buying, holding, or selling bonds. This model was enhanced by Hodges and Schaefer (1977) [16], demonstrating that a deterministic optimization approach could reduce bond portfolio cost and initial investments while maintaining constraints and reinvesting remaining cash. Ronn (1987) [17] further developed this model by integrating bid and ask prices to reflect the investor’s yield curve.

In addressing the complexities of cash flows, interest rates, and obligations, Bradley and Crane (1972) [6] introduced a dynamic multi-period bond portfolio model utilizing a decomposition technique. Sequential decision theory and two-stage programming under uncertainty were employed by Shapiro (1988) [5]. Shapiro’s model incorporated multiple scenarios to address uncertainties in fixed-income portfolio selection.

Korn and Koziol (2006) [18] expanded on the bond portfolio optimization problem, further exploring stochastic programming approaches. Stoyan and Kwon (2011) [19] applied a stochastic-goal mixed-integer programming method to integrate stock and bond portfolios. He and Qu (2014) [20] proposed a two-stage mixed-integer programming model that addressed trading constraints and market uncertainty.

Recent contributions to the field include Maggioni et al. (2020) [21], that provided three mathematical formulations for multi-horizon stochastic programs, and Alreshidi et al. (2020) [14], that introduced new mixed-integer stochastic programming models for bond portfolio optimization in the Saudi bond market. Alkailany et al. (2022) [15] applied the two-stage stochastic programming model developed by Alreshidid et al. (2020) [14] to the U.S. bond market with discrete random variables. A recent comprehensive survey by Salo et al. (2024) [22] highlights both the enduring relevance and the contemporary advances of portfolio optimization, providing context for our contribution to the bond portfolio setting.

Stochastic programming, also known as optimization under uncertainty, is a crucial branch of optimization that addresses problems involving uncertain parameters. In such scenarios, some of the input parameters in the objective function or constraints are random variables, which significantly complicate the decision-making process (see, e.g., Prékopa, 1995 [11]). To manage this uncertainty, two primary approaches are typically employed: analyzing the statistical characteristics of the random parameters or reformulating the problem to account for the joint probability distribution of these parameters. The resulting numerical problem is often complex and requires sophisticated mathematical programming techniques to solve (Prékopa 1995 [11]).

Stochastic programming represents a prominent approach among uncertainty modeling techniques in optimization, offering broad adoptibility and implementation potential across scientific and engineering domains. The integration of probability theory, optimization, statistics, and functional analysis forms the mathematical foundation of stochastic programming methodology. Contemporary computational capabilities and algorithmic developments have facilitated the field’s evolution, enabling increasingly sophisticated modeling frameworks and solution methodologies, as demonstrated through extensive research (Zhang et al., 2023 [23]; Herion and Römisch 2022 [24]; Chan et al. 2025 [25]).

The evolution of stochastic programming in finance traces back to Markowitz’s (1952) [4] seminal work on portfolio selection, which laid the groundwork for optimization under uncertainty. Dantzig and Infanger (1993) [26] extended these concepts to multi-stage stochastic programs for portfolio management. A stochastic network programming approach for financial planning problems was presented by Mulvey and Vladimirou (1992) [27]. Some other applications include fixed-income securities management and asset-liability modeling by Zenios (1995) [28], management of real-world investment portfolios by Carino et al. (1998) [29], optimal portfolio selection by Dentcheva and Ruszczynski (2006) [30], portfolio management by Gülpınar et al. (2016) [31], and methods for incorporating risk-averse approach by Shapiro and Ugurlu (2016) [32]. Following the 2008 financial crisis, Jobst et al. (2009) [33] developed integrated liquidity and market risk models using multi-stage stochastic programming, and Fábán et al. (2011) [34] introduced risk-averse portfolio optimization approaches. Recently, Bertsimas and Kallus (2020) [35] incorporated machine learning techniques within stochastic programming frameworks to enhance predictive accuracy in portfolio optimization.

Hodges and Schaefer (1977) [16] presented a deterministic optimization model to improve the bond portfolio selection performance efficiently. The model minimizes the cost and initial investment of a bond portfolio while constraints are satisfied at each period with the same cash flow pattern, assuming any remaining cash is used for reinvestment in the current period. Later, Ronn (1987) [17] developed a model of bond portfolio based on Hodges and Schaefer’s (1977) [16] findings, introducing the bid and ask price of bond into the model. The proposed model may display an investors yield curve. Bradley et al. (1972) [6] introduced a dynamic model for multi-period bond portfolio optimization and proposed a decomposition technique to address the model’s computational complexity. They explored two major approaches, sequential decision theory and two-stage stochastic programming, to solve multi-asset, two-stage portfolio problems, explicitly incorporating uncertainty in cash flows, interest rates, and financial obligations. As they noted, normative models for such decision problems tend to become significantly large, particularly when the model’s dynamic structure and the stochastic nature of future interest rates and cash flows are fully incorporated. The second approach allows bond portfolio managers to minimize the cost of a bond portfolio taking into account various constraints on the bonds using the historical data (Shapiro 1988 [5]).

A stochastic programming model for fixed income portfolio optimization was developed by Shapiro (1988) [5] to treat model uncertainties. The idea is to cover most of the future expected cases by considering a different number of scenarios. The optimal solution strategy for selling, buying, and/or holding for the bond portfolio is calculated by the proposed model, where cash flows, interest rates, and/or liabilities may have uncertainties (Shapiro 1988 [5]). For more information on the bond portfolio optimization problem, the reader is referred to Korn and Koziol (2006) [18], Alreshidi et al. (2020) [14] and the references therein.

A Stochastic-Goal Mixed-Integer Programming approach for an integrated stock and bond portfolio problem was used by Stoyan and Kwon (2011) [19]. He and Qu (2014) [20] proposed a two-stage mixed-integer programming model for a portfolio selection issue that takes into account a wide range of actual trading constraints in addition to market random uncertainty over asset prices. Complementary to these approaches, Consigli et al. (2025) [36] developed asset-liability management models with sequential stochastic dominance, illustrating the growing focus on risk-aware extensions of stochastic programming. The reader is referred to a recent article by Maggioni et al. (2019) [21] in which three general mathematical formulations of a multi-horizon stochastic program are presented, and definitions of the classical Expected Value problem and Wait-and-See problem are expanded in a multi-horizon framework. Alreshidi et al. (2020) [14] proposed three new mixed-integer stochastic programming models for two-stage bond portfolio optimization and its application on the Saudi Sukuk (bond) market, where an investor may optimize the cost of a bond portfolio under different scenarios. Alkailany et al. (2022) [15] presented a two-stage bond portfolio optimization model and its application in the U.S. market, assuming the random input parameters are independent, identically distributed discrete random variables. Related research has also applied multi-stage stochastic programming to corporate bond issuance, underscoring the growing importance of stochastic methods in fixed-income markets (Yang et al., 2022 [37]). Recent studies emphasize the role of adaptive two-stage stochastic models in large-scale planning contexts, such as capacity expansion (Basciftci et al., 2024 [38]).

In this research, we present a stochastic two-stage bond portfolio model as a modification of a model introduced by Alreshidi et al. (2020) [14]. Unlike the previous study, where the underlying distribution is discrete, we assume that the random variables associated with the interest rate and second-stage obligations follow a continuous distribution. As a result, the problem involves an infinite number of scenarios, making it more complex and realistic in modeling financial uncertainties.

While the model proposed by Alreshidi et al. (2020) [14] serves as a foundational reference, we propose a Monte Carlo Simulation framework, which can effectively handle the computational complexity and is particularly well suited for problems characterized by continuous uncertainty.

The proposed algorithmic framework offers several advantages over prior studies. It enhances the accuracy of scenario generation through continuous probability distributions, leading to more reliable decision-making outcomes. Additionally, the use of Monte Carlo simulation improves computational efficiency in handling high-dimensional uncertainty, making the model both theoretically significant and practically applicable in real-world financial decision-making, particularly in bond portfolio optimization.

The exact origin of using Monte Carlo simulation methods to solve stochastic programming problems is difficult to pinpoint. Rubinstein and Shapiro (1993) [39] covered the stochastic counterparts algorithm, laying foundational concepts for optimization using Monte Carlo methods. Similar ideas were employed by Geyer and Thompson (1992) [40] to compute maximum likelihood estimators through Gibbs sampling-based Monte Carlo approaches. Infanger (1992) [41] developed algorithms combining classical decomposition and Monte Carlo sampling to effectively solve large-scale stochastic linear programs.

It is worth noting that the term “sample average approximation (SAA)” is not uniformly used in the literature. Plambeck et al. (1996) [42] referred to it as “sample-path optimization.” This approach, also known as sample average approximation, is based on Monte Carlo concepts (Gurkan et al., 1994 [43]). Shapiro and Wardi (1996) [44] provided a framework ensuring the convergence of a Monte Carlo-based approximation method with a probability one. Later on, Shapiro and Homem-de-Mello (1998) [45] illustrated the methodology of using a Monte Carlo simulation-based approach on a two-stage stochastic program with recourse, discussing algorithmic details, fundamental principles, and providing several numerical examples. Moreover, the error estimation, stopping rules, and validation analysis were developed by incorporating statistical inference into numerical algorithms (Shapiro 1988 [5]).

Sampling-based approaches have been effectively applied in various stochastic optimization contexts. Notable applications include vehicle routing (Verweij et al., 2023 [46]), engineering design (Royset and Polak 2004 [47]), supply chain network design (Santoso et al., 2005 [48]), generation and transmission (Jirutitijaroen and Singh 2008 [49]), scheduling (Keller and Bayraksan 2009 [50]), and asset management (Hilli et al., 2007 [51]). The most recent survey on Monte Carlo sampling-based approaches for stochastic optimization is discussed by Homem-de-Mello and Bayraksan (2014) [52]. Their work provides a comprehensive overview, offering practical guidance for tackling stochastic optimization problems using sampling methods. Recent advances such as adaptive sequential SAA methods (Pasupathy and Song 2021 [53]) demonstrate how iterative control of sample sizes can improve efficiency. Extensions of the SAA approach have increasingly focused on integrating auxiliary information, such as covariates, to improve sample efficiency (Kannan et al. 2025 [54]), highlighting the ongoing evolution of adaptive sampling methods. Further, a recent work by Lew et al. (2022) [55] examined limitations of the SAA under equality constraints and non-convex problems, reinforcing the need for adaptive strategies.

In the following section, we provide the two mathematical models that will serve as basis for our Monte Carlo simulation approach.

## 3. Basic Mathematical Models for Bond Portfolio Optimization

### 3.1. Bond Portfolio Optimization Problem for Stochastic Programming with Recourse

Our modeling approach builds on the classical two-stage recourse stochastic programming framework (Shapiro 1988 [5]). In this framework, a set of first-stage (here-and-now) decisions is taken before the uncertainty is revealed. After the random outcomes are realized, second-stage (recourse) decisions are made to adjust the initial plan at an additional cost. Formally, the generic two-stage stochastic programming model can be written as

(4) $$\underset{x\in X}{\text{min}}\;c^{⊤}x+E_{\xi }\left[Q\left(x,\xi \right)\right],$$

where *x* represents the first-stage decision vector, *c* is the associated cost, ξ is a random vector of uncertain parameters, and Q(x,ξ) denotes the optimal value of the recourse problem given *x* and realization ξ. This structure provides a flexible foundation for modeling portfolio optimization problems under uncertainty.

In the context of bond portfolio optimization, Shapiro’s recourse stochastic programming model provides a foundation for addressing uncertainties. The model employs the following notations (Shapiro 1988 [5]):

*B*: number of bonds
*T*: number of time periods
$y_{j}$: amount of bond *j* purchased for all j=1,…,B
$c_{j}$: cost of bond *j* for all j=1,…,B
$t^{*}$: last time period when all parameters are known with certainty
$z_{t}$: cash surplus accumulated at the end of the period *t* for all t=0,…,T
$a_{jt}$: cash flows obtained from bond *j* at time *t* for j=1,…,B and t=0,…,T
*K*: number of scenarios of the uncertain future
$\rho_{kt}=1+i_{kt}$: re-investment rate under scenario *k* for period *t*, where $i_{kt}$ is the interest rate for all t=0,…,T and k=1,…,K
$L_{kt}$: cash liability to be met in period *t* under scenario *k* for all t=0,…,T and k=1,…,K
$p_{k}$: probability that scenario *k* will occur for all k=1,…,K
$D_{kt}$: additional cash required in period *t* under scenario *k* for all t=0,…,T and k=1,…,K
$v_{kt}$: discount factor for cash flows in period *t* under scenario *k* for all t=0,…,T and k=1,…,K

Then, problem (5) minimizes the cost of bonds purchased in the first stage and the expected cost of bonds purchased in the second stage under various scenarios, while maximizing the expected gain from selling bonds that have not yet matured. The constraints of problem (5) ensure that obligations are met by cash flows from bonds and reinvested surplus (Shapiro 1988 [5]).

(5) $$\begin{array}{cc} \text{min} & \sum_{j=1}^{B}c_{j}y_{j}+\sum_{k=1}^{K}\sum_{t=t^{*}+1}^{T}p_{k}v_{kt}D_{kt}+z_{0}\\ \text{subject to}\\ \; & \sum_{j=1}^{B}a_{jt}y_{j}+\rho_{t}z_{t-1}-z_{t}=L_{t},\;t=1,\ldots ,t^{*}\\ \; & \sum_{j=1}^{B}a_{jt}y_{j}+\rho_{kt}z_{t-1}-z_{t}+D_{kt}=L_{kt},\;t=t^{*}+1,\ldots ,T,\;k=1,\ldots ,K\\ \; & y_{j}\geq 0,\;z_{t}\geq 0,\;D_{kt}\geq 0,\;k=1,\ldots ,K,\;j=1,\ldots ,N,\;t=0,\ldots ,T \end{array}$$

### 3.2. Two-Stage Mixed-Integer Stochastic Programming Models for Bond Portfolio Optimization

In this section, we consider an investor who aims to secure the required liabilities over a fixed time period through investments in bonds. The investor seeks to develop an optimal investment plan that ensures these liabilities are met through the returns generated from the bond portfolio. To achieve this, the investor needs to make strategic decisions regarding the selection and allocation of bonds, considering both immediate investment opportunities (first-stage) and future adjustments based on market uncertainties (second-stage). This problem naturally leads to the formulation of a two-stage bond portfolio optimization model, where the initial investment decisions are made before the uncertainty is realized, and recourse actions are taken in the second stage to adjust the portfolio in response to observed financial conditions.

Alreshidi et al. (2020) [14] formulated a new bond portfolio optimization model as a two-stage stochastic programming problem. The model’s objective is to maximize the expected gain from selling bonds under various scenarios, defined by interest rates in each period, while minimizing the cost of purchasing bonds in both stages. Alreshidi et al. (2020) [14] formulated the following two-stage bond portfolio optimization model:

(6) $$\begin{array}{c} \mathrm{minimize}\;\;\sum_{j\in N}c_{j}y_{j}+\sum_{k=1}^{K}\sum_{j\in M}p_{k}P_{jk}x_{jk}-\sum_{k=1}^{K}\sum_{j\in N-N^{*}}\sum_{t=t^{*}+1}^{T}p_{k}Q_{jkt}s_{jkt}+z_{0}\\ \mathrm{subject}\;\mathrm{to}\\ \sum_{j\in N-N^{*}}g_{jt}y_{j}+\rho_{t}z_{t-1}-z_{t}=L_{t},\;t=1,\ldots ,t^{*}\\ \;\\ \sum_{j\in N-N^{*}}g_{j(t^{*}+1)}y_{j}-\sum_{j\in N-N^{*}}g_{j(t^{*}+1)}s_{jk(t^{*}+1)}+\psi_{k(t^{*}+1)}\sum_{j\in N-N^{*}}Q_{jk(t^{*}+1)}s_{jk(t^{*}+1)}\\ \;\;\;\;+\sum_{j\in M}f_{jk(t^{*}+1)}x_{jk}+\psi_{k(t^{*}+1)}z_{t^{*}}-h_{k(t^{*}+1)}=L_{k(t^{*}+1)},\;\;k=1,\ldots ,K\\ \;\\ \sum_{j\in N-N^{*}}g_{jt}y_{j}-\sum_{r=t^{*}+1}^{t}\sum_{j\in N-N^{*}}g_{jt}s_{jkr}+\psi_{kt}\sum_{j\in N-N^{*}}Q_{jkt}s_{jkt}\\ \;\;\;\;+\sum_{j\in M}f_{jkt}x_{jk}+\psi_{kt}h_{k(t-1)}-h_{kt}=L_{kt},\;t=t^{*}+2,\ldots ,T,\;k=1,\ldots ,K\\ \;\\ \sum_{t=t^{*}+1}^{T}s_{jkt}\leq y_{j},\;j\in N-N^{*},\;k=1,\ldots ,K\\ y_{j}\leq u_{j},\;j\in N\\ x_{jk}\leq u_{j},\;j\in M,\;k=1,\ldots ,K\\ y_{j}\geq 0,\;y_{j}\in Z,\;j\in N\\ x_{jk}\geq 0,\;x_{jk}\in Z,\;j\in M,\;k=1,\ldots ,K\\ s_{jkt}\geq 0,\;s_{jkt}\in Z,\;j\in N-N^{*},\;t=t^{*}+1,\ldots ,T,\;k=1,\ldots ,K\\ z_{t}\geq 0,\;t=0,\ldots ,t^{*}\\ h_{kt}\geq 0,\;t=t^{*}+1,\ldots ,T,\;k=1,\ldots ,K \end{array}$$

where the input parameters and decision variables are as defined in Table 1 and Table 2, respectively.

The objective function of problem (6) aims to minimize the cost of bonds purchased in the first-stage, $\sum_{j\in N}c_{j}y_{j}$, where $c_{j}$ is the buying price of the bond *j* in Stage 1.

Similarly, it seeks to reduce the expected cost of bonds purchased in the second-stage under scenario *k*, $\sum_{j\in M}p_{k}P_{jk}x_{jk}$, where $P_{jk}$ is the buying price of bond *j* purchased under scenario *k* as defined before, and $p_{k}$ is the probability that scenario *k* will occur. Additionally, it aims to maximize the expected gain from selling bonds not yet matured in Stage 2, $\sum_{j\in N-N^{*}}\sum_{t=t^{*}+1}^{T}p_{k}Q_{jkt}s_{jkt}$, where $Q_{jkt}$ is the selling price of bond *j* under scenario *k* at time *t*, and $p_{k}$ is the probability that scenario *k* will occur.

Overall, the objective is to minimize the initial investment, $z_{0}$, by balancing the costs of bond purchases in both stages and maximizing the expected returns from bond sales under various scenarios.

Note that the first set of equality constraints in problem (6) ensures that for each period $t=1,\ldots ,t^{*}$, the obligation requirements are met by the cash flow from bonds *j* (which have not yet matured) purchased in Stage 1, combined with the surplus from the previous period reinvested in the current period. These constraints are referred to as the liability constraints for Stage 1.

The second set of equality constraints represents the liability constraint at the transition stage $t^{*}+1$, moving from Stage 1 to Stage 2, where $t^{*}$ is the last time period in which all parameters are known with certainty under scenario k=1,…,K. For this period, the liability requirement will be met by holding or selling the bonds owned or buying new bonds only in the first period of the second stage. Note that the first term is the total cash flow obtained from bonds purchased in Stage 1 that have not yet reached maturity. The second term represents the cash flow for bonds sold in the first period in Stage 2. The third and fourth terms are the interest and cash from the sales of the bonds in Stage 2 and cash flow from the bonds purchased in Stage 2. Note also that the cash flow for the transition period uses the cash surplus $z_{t^{*}}$, which is accumulated at the end of the final period of Stage 1 and may be used for the purchase of new bonds in the first period of Stage 2 under any scenario *k*.

The third set of equality constraints is similar to the transition stage constraints. It represents the remaining periods, $t=t^{*}+2,\ldots T$. The main difference is the calculation of cash surplus that will be used to purchase new bonds in the current period of Stage 2, in this case, will be the cash accumulated at the end of the previous period of Stage 2.

The fourth set of constraints ensures that the amount of bond *j* purchased in Stage 1 is greater than the total amount of bond *j* for any $j\in N-N^{*}$ (that has not yet reached the maturity) to be sold in Stage 2 under any scenario *k* at the beginning of the period $t=t^{*}+1,\ldots ,T$. The integrality constraints in problem (6) have been added for the amount of bonds that may be purchased or sold within the planning horizon in addition to the liability constraints. We set an upper bound for the amount of bond *j* that can be purchased in Stage 1 or Stage 2.

A thorough examination of problem (6) proposed by Alreshidi et al. (2020) [14] leads to the observation that the amount of funds resulting from selling bonds in Stage 2 is subtracted from the objective function (cost of bond portfolio); on the other hand, those funds are used to meet liabilities constraints. Therefore, to respect accounting laws, we propose to modify problem (6) by replacing the second and third set of liability constraints with the following constraints, where reinvestment rates, $\psi_{kt}$, are replaced with interest rates, $r_{kt}$, under scenario *k* and for periods $t=t^{*}+1,\ldots ,T$ in Stage 2:

(7) $$\begin{array}{c} \sum_{j\in N-N^{*}}g_{j(t^{*}+1)}y_{j}-\sum_{j\in N-N^{*}}g_{j(t^{*}+1)}s_{jk(t^{*}+1)}+r_{k(t^{*}+1)}\sum_{j\in N-N^{*}}Q_{jk(t^{*}+1)}s_{jk(t^{*}+1)}\\ \;\;\;\;\;+\sum_{j\in M}f_{jk(t^{*}+1)}x_{jk}+\psi_{k(t^{*}+1)}z_{t^{*}}-h_{k(t^{*}+1)}=L_{k(t^{*}+1)},\;k=1,\ldots ,K \end{array}$$

(8) $$\begin{array}{c} \sum_{j\in N-N^{*}}g_{jt}y_{j}-\sum_{r=t^{*}+1}^{t-1}\sum_{j\in N-N^{*}}g_{jt}s_{jkr}+r_{kt}\sum_{j\in N-N^{*}}Q_{jkt}s_{jkt}\;\;\;\;\;\;\;\;\;\\ \;\;\;\;\;\;\;\;\;+\sum_{j\in M}f_{jkt}x_{jk}+\psi_{kt}h_{k(t-1)}-h_{kt}=L_{kt},\;\;t=t^{*}+2,\ldots ,T \end{array}$$

This results in the following two-stage bond portfolio optimization model:

(9) $$\begin{array}{c} \text{minimize}\;\;\sum_{j\in N}c_{j}y_{j}+\sum_{k=1}^{K}\sum_{j\in M}p_{k}P_{jk}x_{jk}-\sum_{k=1}^{K}\sum_{j\in N-N^{*}}\sum_{t=t^{*}+1}^{T}p_{k}Q_{jkt}s_{jkt}+z_{0}\\ \text{subject}\;\text{to}\\ \sum_{j\in N-N^{*}}g_{jt}y_{j}+\rho_{t}z_{t-1}-z_{t}=L_{t},\;t=1,\ldots ,t^{*}\\ \sum_{j\in N-N^{*}}g_{j(t^{*}+1)}y_{j}-\sum_{j\in N-N^{*}}g_{j(t^{*}+1)}s_{jk(t^{*}+1)}+r_{k(t^{*}+1)}\sum_{j\in N-N^{*}}Q_{jk(t^{*}+1)}s_{jk(t^{*}+1)}\\ \;\;\;\;+\sum_{j\in M}f_{jk(t^{*}+1)}x_{jk}+\psi_{k(t^{*}+1)}z_{t^{*}}-h_{k(t^{*}+1)}=L_{k(t^{*}+1)},\;k=1,\ldots ,K\\ \sum_{j\in N-N^{*}}g_{jt}y_{j}-\sum_{r=t^{*}+1}^{t}\sum_{j\in N-N^{*}}g_{jt}s_{jkr}+r_{kt}\sum_{j\in N-N^{*}}Q_{jkt}s_{jkt}\\ \;\;\;\;+\sum_{j\in M}f_{jkt}x_{jk}+\psi_{kt}h_{k(t-1)}-h_{kt}=L_{kt},\;t=t^{*}+2,\ldots ,T,\;k=1,\ldots ,K\\ \sum_{r=t^{*}+1}^{T}s_{jkt}\leq y_{j},\;j\in N-N^{*}\;k=1,\ldots ,K\\ y_{j}\leq u_{j},\;j\in N\\ x_{jk}\leq u_{j},\;j\in M,\;k=1,\ldots ,K\\ y_{j}\geq 0,\;y_{j}\in Z,\;j\in N\\ x_{jk}\geq 0,\;x_{jk}\in Z,\;j\in M,\;k=1,\ldots ,K\\ s_{jkt}\geq 0,\;s_{jkt}\in Z,\;j\in N-N^{*},\;t=t^{*}+1,\ldots ,T,\;k=1,\ldots ,K\\ z_{t}\geq 0,\;t=0,\ldots ,t^{*}\\ h_{kt}\geq 0,\;t=t^{*}+1,\ldots ,T,\;k=1,\ldots ,K \end{array}$$

where input parameters and decision variables are as defined in Table 1 and Table 2, respectively.

## 4. A Monte Carlo Simulation Approach to Solve Problem (9)

The Monte Carlo simulation method is a powerful tool for solving stochastic programming problems. This method involves using random sampling to approximate mathematical functions and simulate the behavior of complex systems. Its flexibility and robustness make it particularly well suited for optimization problems that involve uncertain or random elements. The basic idea of Monte Carlo simulation in the context of stochastic optimization is to generate a large number of random samples from the input distribution, evaluate the objective function for each sample, and use these estimates to guide the search for the optimal solution. This approach is especially useful when the expectation function in a stochastic programming model cannot be calculated accurately or efficiently.

We propose a Monte Carlo simulation-based approach to solve problem (9). Our method is motivated by Algorithm 1 developed by Homem-de-Mello and Bayraksan (2014) [52] that employs sampling methods in stochastic optimization as outlined below:
**Algorithm 1** Sample Average Approximation Framework (Homem-de-Mello and Bayraksan 2014 [52])  **Initialization:** Choose an initial guess $x^{0}$, and set iteration counter k=1.  **Sampling:** Obtain a realization $\left\{{\hat{\xi }}^{k,1},{\hat{\xi }}^{k,2},\ldots ,{\hat{\xi }}^{k,N_{k}}\right\}$ of $\xi^{1},\xi^{2},\ldots ,\xi^{N_{k}}$.  **Optimization:** Perform optimization steps on the function$${\hat{g}}_{Nk}\left(x,\left\{{\hat{\xi }}^{k,1},{\hat{\xi }}^{k,2},\ldots ,{\hat{\xi }}^{k,N_{k}}\right\}\right)$$  possibly using information from previous iterations to obtain $x^{k}$.  **Convergence:** Check the stopping criteria; if not satisfied, then set k:=k+1 and return to Step 2.

Algorithm 1 can be viewed as a general framework that allows for numerous variations of the sample average approximation (SAA) approach (Homem-de-Mello and Bayraksan 2014 [52]). To develop a variety of SAA algorithms, several key questions must be addressed, including determining the sample size for each iteration, generation of realizations from previous iterations, selection of a method for the solution of the underlying optimization problem, and determining the stopping criteria. To approximate the expectation in the stochastic objective function, we apply the SAA technique. From an information-theoretic perspective, each scenario sampled represents a discrete state of nature that contributes to the overall uncertainty in the model. By generating a sufficiently large and diverse set of scenarios, we aim to approximate the true probability distribution of market behaviors.

To solve problem (9), we propose to modify Algorithm 1 as described in Algorithm 2:
**Algorithm 2** Modified Sample Average Approximation Framework to Solve Two Stage Problems**Initialization:** Set $k=1,N_{k}=\bar{N}$, and $R=\bar{R}$, where $\bar{N}$ and $\bar{R}$ are initial values of sample size and number of realizations, respectively.**Sampling:** Generate *R* different realizations, each with *k* different samples$$\left\{\xi_{1}^{1},\xi_{2}^{1},\ldots ,\xi_{N_{k}}^{1}\right\},\left\{\xi_{1}^{2},\xi_{2}^{2},\ldots ,\xi_{N_{k}}^{2}\right\},\ldots ,\left\{\xi_{1}^{R},\xi_{2}^{R},\ldots ,\xi_{N_{k}}^{R}\right\}$$**Optimization:** Compute ${\hat{f}}_{N_{k}}^{1}$, *…*,${\hat{f}}_{N_{k}}^{R}$ and solve the underlying integer linear program using deterministic optimization techniques.**Estimation:** Compute the average optimal cost and its variance:$${\bar{f}}_{k}=\frac{1}{R}\sum_{r=1}^{R}{\hat{f}}_{N_{k}}^{r}$$$$\sigma_{f_{k}}^{2}=\frac{1}{R-1}\sum_{r=1}^{R}\left({\hat{f}}_{N_{k}}^{r}-{\bar{f}}_{N_{k}}\right)$$**First Convergence Check:** If $H_{0}:{\bar{f}}_{k}={\bar{f}}_{k-1}$ is accepted, then go to Step 6. Otherwise, go to Step 7.**Second Convergence Check:** If $H_{0}:{\sigma^{2}}_{f_{k}}=\sigma_{f_{k-1}}^{2}$ is accepted, then go to Step 8. Otherwise, go to Step 7.**Looping:** Set $k=k+1,N_{k}=ck$, where *c* is the number of increments for each iteration, and return to Step 2.**Upper and Lower Bounds:** Compute an upper bound (UB) and a lower bound (LB) for each candidate solution, and select the best solution based on the smallest optimality gap between UB and LB.

The main idea behind determining the sample size is to choose the minimum number of samples that provide a highly accurate approximation. Although larger sample sizes yield better approximations, they also increase computational costs. Our proposed methodology computes the sample size $N_{k}$ at each iteration as $N_{k}=ck$ for each k>0, where $N_{0}$ and *c* are constants (input parameters). More details about the sample size and an investigation of the rate of convergence can be found in Homem-de-Mello and Bayraksan (2014) [52].

For Algorithm 2, we propose to generate a new set of realizations $\left\{\xi^{1},\xi^{2},\ldots ,\xi^{N_{k}}\right\}$ at each iteration instead of extending the realizations from the previous iteration.

As for the stopping criteria, we propose two methods, one related to the average cost and the other to the standard deviation of the cost.

For the first criterion, Algorithm 2 stops based on the result of the following statistical test:

$$H_{0}:{\bar{f}}_{k}={\bar{f}}_{k-1},\;H_{a}:{\bar{f}}_{k}<{\bar{f}}_{k-1}$$

For the second criterion, related to the standard deviation of the cost, Algorithm 2 uses the following statistical test:

$$H_{0}:\sigma^{2}f_{k}=\sigma^{2}f_{k-1},\;H_{a}:\sigma^{2}f_{k}<\sigma^{2}f_{k-1}$$

Algorithm 2 continues iterating until both null hypotheses are accepted, indicating that further iterations are unlikely to result in significant improvements.

To select the best solution among *R* candidate solutions delivered in the last iteration of the proposed algorithm, we assess an upper bound and a lower bound for each candidate solution and consider the solution with the smallest optimality gap. For the selection of optimal solutions, we adopt a framework similar to the one used by Alnaggar et al. (2020) [56] for solving a two-stage transportation problem.

Let ($y_{r},z_{r}$) denote the vector solution of the first-stage problem for the $r^{\mathrm{th}}$ candidate solution and $\eta_{r}$ denote its objective function value. To determine a lower bound of the objective function value, we start by computing the average $\bar{\eta }$ and the variance $\sigma_{N,R}^{2}$ of the objective function values $\eta_{1},\ldots ,\eta_{R}$.

(10) $$\bar{\eta }=\frac{1}{R}\sum_{r=1}^{R}\eta^{r}$$

(11) $$\sigma_{N,R}^{2}=\frac{1}{\left(R-1\right)}\sum_{r=1}^{R}{\left(\eta^{r}-\bar{\eta }\right)}^{2}.$$

Then the lower bound can be obtained as:

(12) $$LB=\bar{\eta }-Z_{\alpha }\frac{1}{\sqrt{R}}\sigma_{N,R}$$

where $Z_{\alpha }$ is the α critical value of the standard normal distribution, given that R≥30.

The upper bound of the true objective function for each potential solution is determined by examining the solution using a large scenario tree of size $N^{\prime }$, assumed to accurately represent the actual cost distribution. Since each scenario $k\in \{1,2,\ldots ,N^{\prime }\}$ is an independent, identically distributed (i.i.d.) random sample, the task of assessing a candidate solution breaks down into $N^{\prime }$ sub-problems. The size of the scenario tree $N^{\prime }$ significantly exceeds the size of the scenario tree handled in the last iteration of Algorithm 2, $N_{K}$, where *K* is the index of the last iteration of the algorithm.

We represent the objective function value, $\varphi_{i}\left(y^{r},z^{r},i\right)$, of a specific sub-problem *i* as:

(13) $$\varphi \left(y^{r},z^{r},i\right)=\sum_{j\in M}P_{ji}x_{ji}+\sum_{j\in N-N^{*}}\sum_{t=t^{*}+1}^{T}p_{k}Q_{jit}s_{jit}\;\text{for all}\;i\in \left\{1,2,\ldots ,N^{\prime }\right\}.$$

It is important to highlight that, since each subproblem is addressed independently, a large value for $N^{\prime }$ does not impose a substantial computational load.

The estimation of the objective function value of the second-stage problem, denoted as $\varphi (y^{r},z^{r})$, is calculated by:

(14) $$\varphi \left(y^{r},z^{r}\right)=\frac{1}{\left(N^{\prime }\right)}\sum_{i=1}^{N^{\prime }}\varphi_{i}\left(y^{r},z^{r},i\right)\;\text{for all}\;i\in \left\{1,2,\ldots ,N^{\prime }\right\}.$$

The value of the objective function ${\bar{\eta }}^{r}$ and its variance $\sigma_{N^{\prime }}^{2}\left(y^{r},z^{r}\right)$ for candidate solution ($y^{r},z^{r}$) are computed as follows:

(15) $${\bar{\eta }}^{r}=\sum_{j\in N}c_{j}y_{j}^{r}+\varphi \left(y^{r},z^{r}\right)+Z_{0}^{r}$$

(16) $$\sigma_{N^{\prime }}^{2}\left(y^{r},z^{r}\right)=\frac{1}{\left(N^{\prime }-1\right)}\sum_{i=1}{\left[\left(\varphi_{i}\left(y^{r},z^{r},i\right)\right)-\varphi \left(y^{r},z^{r}\right)\right]}^{2}.$$

Finally, the upper bound, $UB_{r}$, of a candidate solution can be computed as:

(17) $${UB}_{r}={\bar{\eta }}^{r}+\frac{1}{\left(\sqrt{N^{\prime }}\right)}Z_{\alpha }\sigma_{N^{\prime }}\left(y^{r},z^{r}\right)$$

where $Z_{\alpha }$ is the α critical value of the standard normal distribution. The upper bound, UB, delivered by Algorithm 2 is the smallest value, $UB_{r}$ for all r∈{1,2,…,R}, as defined in Equation (18). The solution that results in the smallest optimality gap, $\left(UB_{r}-LB\right)$ for all candidate solutions, r∈{1,2,…,R}, is the final solution, ($y^{*},z^{*}$ ), of the SAA, which corresponds to the solution with the smallest upper bound $UB_{r}$, is as shown in Equation (19).

(18) $$UB=\min_{r\in 1,\ldots ,R}\left(UB_{r}\right)$$

(19) $$\left(y^{*},z^{*}\right)={\mathrm{argmin}}_{r\in 1,\ldots ,R}\left(UB_{r}\right)$$

## 5. Case Study: Monte Carlo Simulation for Two-Stage Bond Portfolio Optimization in the Saudi Sukuk Market

In this section, we apply Algorithm 2 to solve problem (9) using data from the Saudi Sukuk (bond) market. This case study demonstrates the practical implementation and effectiveness of the proposed methodology in a real-world financial context.

The data for this case study are sourced from the Saudi Stock Exchange (Tadawul) Annual Report of 2011, which provides detailed information on various tradable Sukuk and bonds (Afshar 2013 [57]; Alswaidan 2013 [58]; Alreshidi et al., 2020 [14]).

We select nine bonds for analysis, including SABIC 1, SABIC 2, SABIC 3, SIPCHEM, Saudi Electricity 1, Saudi Electricity 2, Saudi Electricity 3, SATORP, and Saudi Hollandi Bank 2. Key input parameters are summarized below (see Alreshidi et al., 2020 [14] for more details):

− Issue size ranges from Saudi Riyal (SAR) 725 million for Saudi Hollandi Bank 2 to SAR 8000 million for SABIC 2, with a total of SAR 41,274 million.
− Par value varies between SAR 10 thousand for SABIC 2, SABIC 3, SE 3 and SAR 500,000 for Saudi Electricity 1.
− Maturity date varies, earliest with 6 July 2016 for SIPCHEM and latest 10 May 2030 for Saudi Electricity 3.
− Coupon rates spread between SIBOR for Saudi Hollandi Bank 2 and SIBOR + 1.75% for SIPCHEM.
− There is a total of 49 transactions, ranging from 2 for SABIC 1 and SABIC 3 to 14 for Saudi Electricity 2, while SATORP reported no transactions.
− Value traded ranges from SAR 1097.4 thousand for SABIC 3 to about SAR 1.5 billion for Saudi Electricity 3, with a total of over SAR 1.8 billion.
− Nominal value traded ranges from SAR 1.1 million for SABIC 3 to SAR 1.45 milion for Saudi Electricity 3, with a total of over SAR 1.8 billion.
− Stage 1 interest rates at time periods *t* are 0.0148, 0.0317, 0.0112, 0.0112 for t=1,2,3,4, respectively.

The following assumptions are made for the bond portfolio optimization model and calculations of the input parameters are as presented by Alreshidi et al. (2020) [14]:

The total planning horizon is T=10 years, where the first stage covers $t^{*}=4$ years, while the second stage covers the remaining 6 years. We start with K=50 scenarios at each realization and increment by 30 at each iteration until the stopping criteria are met.

The interest rate, $i_{t}$ at time *t*, in both stages is calculated as SIBOR+A%, where SIBOR, the Saudi Interbank Offered Rate, is the benchmark interest rate used by banks in Saudi Arabia for lending to each other and is published daily by the Saudi Arabian Monetary Authority (SAMA), and *A* is a random variable generated uniformly in the range [−2%,2%].

SABIC 1, SABIC 2, SABIC 3, SIPCHEM are considered to be Stage 1 bonds, and Saudi Electricity 1, Saudi Electricity 2, Saudi Electricity 3, SATORP, and Saudi Hollandi Bank 2 are considered to be Stage 2 bonds.

The cost, $c_{j}$, of a bond *j* in Stage 1 and Stage 2 is considered the same as the par value in the Saudi data. Costs for SABIC 1, SABIC 2, SABIC 3, and SIPCHEM bonds are SAR 50,000, SAR 10,000, SAR 10,000, and SAR 100,000, respectively. Costs for Saudi Electricity 1, Saudi Electricity 2, Saudi Electricity 3, SATORP, and Saudi Hollandi Bank 2 bonds are SAR 500,000, SAR 100,000, SAR 10,000, SAR 100,000, and SAR 100,000, respectively.

The buying price, $P_{jk}$, of a bond j∈M under scenario k=1,…,K in the second-stage is calculated as $P_{jk}=\nu_{k}*c_{j}$, where $c_{j}$ represents the purchase price of bond j∈M, which corresponds to its nominal buying price. Additionally, $\nu_{k}$ serves as an impact factor for each scenario *k*, where k=1,…,K, in Stage 2.

The selling price, $Q_{jkt}$, of a bond $j\in N-N^{*}$ in Stage 2 under scenario k=1,…,K for period $t=t^{*}+1,\ldots ,T$ is obtained as follows:

(20) $$Q_{jkt}=\left\{\begin{array}{ccc} \frac{c_{j}}{\prod_{r=1}^{t^{*}}\rho_{r}\prod_{r=t^{*}+1}^{t-1}\psi_{kr}}, & \;\;\;\; & j\in N\\ \frac{P_{jk}}{\prod_{r=t^{*}+1}^{t-1}\psi_{kr}}, & \;\;\;\; & j\in M \end{array}\right.$$

where all parameters are as defined in Table 1.

The cash flow, $g_{jt}$, for bond j∈N in period $t=1,\ldots ,t^{*}$ in Stage 1 is calculated as below:

(21) $$g_{jt}=\left\{\begin{array}{ccc} c_{j}*I_{j} & \;\; & t=1,\ldots ,M_{j}-1\\ c_{j}*\left(1+I_{j}\right) & \;\; & t=M_{j}\\ 0 & \;\; & t>M_{j} \end{array}\right.$$

where $c_{j}$ denotes the purchase price of bond j∈N in Stage 1, $I_{j}$ represents the coupon rate, and $M_{j}$ signify the maturity date of any bond j∈N∪M. The cash flows for Stage 1 bonds, calculated using Equation (21), indicate that SABIC 1, SABIC 2, SABIC 3, and SIPCHEM generate annual amounts of SAR 1575, SAR 313, SAR 323, and SAR 4500, respectively, which remain constant over the four-year horizon.

The cash flows, $f_{jkt}$, for each bond j∈M in Stage 2 under scenario k=1,…,K are calculated using the equation below:

(22) $$f_{jkt}=\left\{\begin{array}{ccc} P_{jk}*I_{j} & \;\; & t=t^{*}+1,\ldots ,M_{j}-1\\ P_{jk}*\left(1+I_{j}\right) & \;\; & t=M_{j}\\ 0 & \;\; & t>M_{j} \end{array}\right.$$

where $P_{jk}$ represent the purchase price of bond *j* acquired in Stage 2 under scenario *k* and $I_{j}$ and $M_{j}$ represent the coupon rate and the maturity date of bond *j* in Stage 2, respectively.

Stage 1 liabilities, $L_{t}$, $t=1,\ldots ,t^{*}$, are a percentage (randomly chosen in the interval [2%, 5%]) of the total cash flows from available bonds at the beginning of Stage 1, where $L_{2}$ = 1,009,895, $L_{3}$ = 2,275,167, $L_{4}$ = 3,064,511, and $L_{5}$ = 4,086,015.

Stage 2 liabilities, $L_{kt},k=1,\ldots ,K,t=t^{*}+1,\ldots \ldots ,T$, are randomly chosen in the interval [0.9B, 1.1B] for the second stage, where *B* is a percentage (randomly chosen in the interval [0.2%, 2%] of the total cash flows from available bonds at the beginning of the second stage.

After solving the two-stage bond-portfolio optimization problem (9) using the Monte Carlo simulation-based approach described in Algortihm 2, we obtain the optimal costs for the Saudi Sukuk (bond) data. This is achieved by implementing the algorithm in C++ with CPLEX. The optimal costs of the two-stage bond-portfolio optimization problems for all realizations and different iterations of the algorithm are presented in Table 3, Table 4 and Table 5.

Table 6 presents the average cost and variance across different iterations. For each iteration, R=30 realizations were considered, and the average cost and variance were computed based on these 30 realizations.

**Figure 1:**
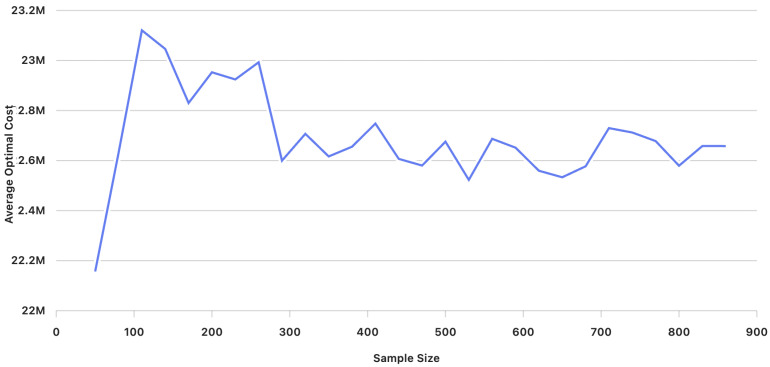
Behavior of the average optimal cost (in Saudi Riyal SAR) of model (6) for sample sizes (number of scenarios) K=50 to K=860.

**Figure 2:**
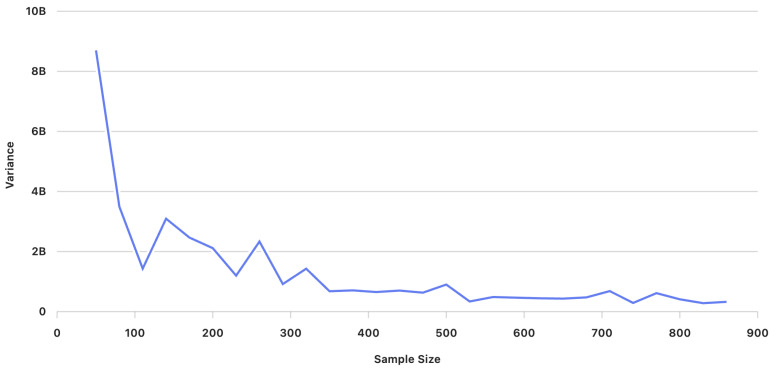
Behavior of the variance of optimal costs (in Saudi Riyal SAR) of model (6) for sample sizes (number of scenarios) K=50 to K=860.

Figure 1 illustrates the average cost for different sample sizes (number of scenarios) K=50 to K=860. We observe that, as the sample size (number of scenarios) increases, the average costs stabilize, with the values converging closer to each other. Additionally, Figure 2 shows the variance of the average cost, revealing that variance decreases as the sample size (number of scenarios) increases.

At each iteration, the statistical tests for both hypotheses were conducted based on the computed sample averages and variance estimates obtained through Algorithm 2. Table 6 presents the average cost (in Saudi Riyal SAR) and variance for different sample sizes (number of scenarios) ranging from K=50 to K=860 scenarios for problem (9).

Based on the results obtained for problem (9) shown in Table 7, we observe that as the number of scenarios, *K*, increases, the average cost initially fluctuates, with noticeable differences in earlier iterations. This variation suggests that the optimization process is still adjusting to increase sample sizes. However, as the number of scenarios, *K*, continues to grow, the differences in mean and variance become statistically insignificant since p>0.05, especially after K=860 scenarios. The F-statistic confirms variance stabilization, indicating that cost fluctuations across realizations have reached a consistent state. At K=860 the stopping criterion is met, as both the T-statistic and F-statistic show that further increases in the number of scenarios, *K*, will not yield statistically significant improvements in cost reduction or stability. Hence, additional iterations would not be computationally efficient, and therefore, $K^{*}=860$, is the optimal stopping sample size.

To investigate the optimal cost, we focus on the optimal sample size, $K^{*}=860$, representing the optimal number of scenarios. To select the best solution among the 30 realizations, we calculate the lower bound, LB, in Equation (12) and the upper bound, UB, in Equation (18) to identify the smallest optimality gap, as described in Section 4. Table 8 indicates that the smallest gap among the 30 realizations is 1.1438, with the optimality range between [SAR 22,652,406 and SAR 22,914,500], where SAR is Saudi Riyal.

Based on the chosen solution, we determine the bonds to be purchased in Stage 1. Specifically, 3979 units of SIPCHEM and 478 units of SABIC 3 are purchased in period 1 of Stage 1, as shown in Table 9.

Table 10 presents the optimal investment plan for Stage 1, based on the modified Model. The total optimal cost of the investment plan, according to the modified Model 1 is SAR 22,637,904.

Table 11 illustrates the computational efficiency of Algorithm 2 based on varying sample sizes (number of scenarios). All computational experiments were carried out on a desktop with Intel(R) Core (TM) i74930K CPU 3.4 GHz processor with 16 GB of memory under a Windows environment. As expected, the computation time increases as the number of scenarios increases. We observe that the computational cost has increased by 50 times when the sample size increases from 1000 scenarios to 2000 scenarios, whereas it significantly decreases for 5000 scenarios. We remark that optimization solvers dynamically adjust their internal methods based on the input data. The decrease in computational cost for the problem with 2000 scenarios to the problem with 5000 scenarios may be a result of more efficient numerical methods or multithreading optimization that are triggered at K=5000 scenarios but were not activated at K=2000 scenarios.

In summary, the results of applying Algorithm 2 along with the proposed stopping criteria to the Saudi Sukuk (bond) data demonstrate the proposed method’s effectiveness and computational efficiency in solving large-scale stochastic optimization problems. The proposed algorithm, Algorithm 2, showcases remarkable computational efficiency.

## 6. Conclusions

This paper presents a Monte Carlo simulation-based approach for solving two-stage stochastic programming problems, specifically applied to bond portfolio optimization. By leveraging random sampling techniques, the proposed method effectively transforms the stochastic problem into a deterministic one, enabling the use of standard optimization techniques. The application of this approach to the Saudi Sukuk (bond) market provides valuable insights and demonstrates the practicality and robustness of the methodology.

The key contributions of this research are many-fold: (1) The integration of Monte Carlo simulation with two-stage stochastic programming offers a novel and efficient way to address uncertainty in bond portfolio optimization. This approach allows for accurate approximation of expectation functions and effective scenario management. (2) The robustness of the approach in dealing with market uncertainties ensures that optimized portfolios remain resilient across different scenarios. (3) The proposed algorithm showcases remarkable computational efficiency and manages to handle large scenario trees and deliver solutions in a reasonable time frame. This efficiency is vital for practical applications, where timely decision-making is crucial. (4) The optimization results indicate substantial cost reductions and effective liability management. Algorithm 2 consistently identifies optimal bond portfolios across various realizations and iterations. By employing the Monte Carlo simulation approach, Algorithm 2 converts the stochastic problem into a deterministic one, making it feasible to solve with standard optimization techniques. (5) One of the significant strengths of the Monte Carlo simulation approach is its ability to handle market uncertainties. The scenario analysis reveals that Algorithm 2 can effectively optimize bond portfolios under varying interest rates and liability conditions. The robustness of the proposed method ensures that the optimized portfolios remain resilient against different market scenarios, thereby minimizing risks and maximizing returns. (6) To ensure the reliability of the results, statistical tests are employed to validate the convergence of the algorithm. The use of sample average approximation (SAA) allows for accurate approximation of the expectation function. The convergence criteria, based on statistical significance tests for average costs and variances, confirm the stability and precision of the solutions. The upper and lower bounds computed for each candidate solution further are validated by the optimality of the results. (7) The practical application of this methodology to the Saudi Sukuk (bond) market demonstrates its real-world relevance. The case study highlights the adaptability of the Monte Carlo simulation approach to different financial instruments and market conditions. The results provide valuable insights for investors and portfolio managers, enabling them to make informed decisions that optimize bond portfolios while effectively managing risks. (8) Incorporating uncertainties such as fluctuating interest rates and liabilities in bond portfolio optimization, the proposed approach allows for better risk management and more resilient investment strategies. The methodology can be applied to other financial markets and instruments, making it a versatile tool for global investment strategies.

## Figures and Tables

**Table 1:** Input data (parameters) for problems (6) and (9).

*N*	Set of bonds purchased in Stage 1
*M*	Set of bonds that can be purchased in Stage 2
*T*	Number of time periods
$t^{*}$	Last time period when all parameters are known with certainty
$N^{*}$	Set of bonds purchased in Stage 1, whose maturity is ≤$t^{*}$, and $N^{*}\subseteq N$
*K*	Number of scenarios in Stage 2
$c_{j}$	Buying price of bond *j* where j∈N∪M
$g_{jt}$	Cash flow obtained from bond *j* in Stage 1 at time *t*, where
	j∈N and t=1,…,T
$f_{jkt}$	Cash flow obtained from bond *j* in Stage 2 under scenario *k* at time *t*,
	where j∈M, k=1,…,K, and $t=t^{*}+1,\ldots ,T$
$L_{t}$	Liability to be met in period *t*, where $t=1,\ldots ,t^{*}$
$L_{kt}$	Liability to be met in period *t* under scenario *k*, where
	$t=t^{*}+1,\ldots ,T$ and k=1,…,K
$i_{t}$	Interest rate in period *t* in Stage 1, where $t=1,\ldots ,t^{*}$
$r_{kt}$	Interest rate in period *t* in Stage 2 under scenario *k*, where
	$t=t^{*}+1,\ldots ,T$ and k=1,…,K
$\rho_{t}$	Re-investment rate in period *t* in Stage 1, where $t=1,\ldots ,t^{*}$ and
	$\rho_{t}$ = 1 + $i_{t}$ for $t=1,\ldots ,t^{*}$
$\psi_{kt}$	Re-investment rate under scenario *k* in period *t* in Stage 2 defined by
	$\psi_{kt}$ = 1 + $r_{kt}$, where $r_{kt}$ is the interest rate for $t=t^{*}+1,\ldots ,T$ and k=1,…,K
$P_{jk}$	Buying price of bond *j* purchased under scenario *k* for j∈M and k=1,…,K
$Q_{jkt}$	Selling price of bond *j* under scenario *k* in period *t*, where
	$j\in N-N^{*}$, $t=t^{*}+1,\ldots ,T$, and k=1,…,K
$p_{k}$	Probability that scenario *k* will occur for k=1,…,K

**Table 2:** Decision variables for problems (6) and (9).

$y_{j}$	Number of units purchased from bond j for j∈N
$z_{0}$	Initial cash investment
$z_{t}$	Cash surplus accumulated at the end of the period t for $t=1,\ldots ,t^{*}$
$h_{kt}$	Cash surplus accumulated at the end
	of the period t under scenario k, $t=t^{*}+1,\ldots ,T$, k=1,…,K
$x_{jk}$	Amount of bond j purchased in Stage 2 under scenario *k* for j∈M
	and k=1,…,K
$s_{jkt}$	Amount of bond *j* sold in Stage 2 under scenario *k* which has not reached the
	maturity where $j\in N-N^{*}$, k=1,…,K and $t=t^{*}+1,\ldots ,T$

**Table 3:** Optimal cost (in Saudi Riyal SAR) of model (9) based on Algorithm 2 for K=50 to K=860 scenarios and R=1 to R=10 realizations.

Sample Size	1	2	3	4	5	6	7	8	9	10
50	22,265,504	22,196,684	22,069,286	22,262,364	22,068,184	22,247,162	22,140,010	22,226,344	22,262,274	22,054,840
80	22,720,722	22,668,642	22,654,792	22,626,936	22,674,966	22,609,232	22,670,382	22,641,696	22,568,942	22,669,854
110	23,138,850	23,180,676	23,118,006	23,139,992	23,144,204	23,117,254	23,157,620	23,198,066	23,043,918	23,059,832
140	23,008,830	23,005,314	23,028,244	23,001,990	23,098,344	23,021,378	22,947,118	23,035,022	23,108,210	23,047,234
170	22,937,088	22,882,234	22,829,972	22,866,132	22,785,988	22,802,760	22,899,402	22,805,734	22,760,046	22,834,454
200	22,979,682	22,865,768	22,963,866	22,882,192	22,969,268	22,989,086	22,986,118	22,895,440	22,914,030	23,043,084
230	22,922,574	22,989,214	22,991,872	22,910,998	22,957,744	22,914,236	22,972,384	22,888,552	22,866,894	22,879,940
260	22,908,240	23,007,658	22,973,378	22,953,190	23,001,674	23,053,246	22,998,946	23,059,180	22,918,848	22,982,410
290	22,634,194	22,593,962	22,619,662	22,590,708	22,613,906	22,627,852	22,558,360	22,582,824	22,617,492	22,634,068
320	22,660,904	22,664,612	22,718,868	22,787,006	22,673,512	22,673,818	22,695,040	22,742,574	22,722,564	22,696,608
350	22,601,522	22,584,608	22,609,338	22,614,594	22,589,642	22,632,240	22,605,740	22,665,256	22,641,672	22,649,372
380	22,657,658	22,624,844	22,629,336	22,594,900	22,646,900	22,650,932	22,658,058	22,653,232	22,654,858	22,657,256
410	22,740,196	22,742,622	22,730,776	22,778,622	22,726,898	22,759,446	22,752,096	22,720,734	22,760,496	22,713,450
440	22,569,322	22,621,648	22,603,778	22,535,884	22,644,062	22,590,278	22,621,280	22,587,942	22,612,952	22,618,754
470	22,591,812	22,626,538	22,566,262	22,552,222	22,626,832	22,560,730	22,583,496	22,611,746	22,570,586	22,547,362
500	22,676,318	22,709,658	22,686,070	22,644,946	22,667,304	22,678,936	22,689,300	22,635,984	22,696,702	22,651,734
530	22,528,930	22,503,006	22,524,058	22,515,074	22,524,546	22,521,206	22,485,202	22,517,848	22,511,472	22,544,572
560	22,700,222	22,658,356	22,718,134	22,723,510	22,703,158	22,663,254	22,657,612	22,692,910	22,700,344	22,650,430
590	22,649,722	22,676,452	22,681,334	22,642,650	22,663,334	22,650,864	22,611,072	22,680,096	22,646,852	22,644,194
620	22,478,812	22,565,170	22,558,228	22,561,604	22,550,718	22,577,476	22,552,456	22,595,662	22,589,688	22,566,446
650	22,547,566	22,535,956	22,549,032	22,550,790	22,560,924	22,509,826	22,540,026	22,508,774	22,517,570	22,506,856
680	22,611,406	22,581,738	22,553,906	22,561,968	22,583,046	22,572,246	22,555,564	22,595,864	22,592,712	22,585,836
710	22,742,404	22,685,252	22,719,710	22,738,206	22,746,072	22,757,838	22,729,038	22,682,084	22,725,248	22,765,626
740	22,694,538	22,712,736	22,724,896	22,684,264	22,703,856	22,738,444	22,715,300	22,733,398	22,685,980	22,736,340
770	22,684,796	22,692,146	22,682,390	22,701,930	22,673,552	22,651,052	22,668,386	22,666,438	22,661,612	22,691,850
800	22,592,514	22,554,728	22,577,354	22,569,026	22,633,540	22,559,830	22,591,684	22,586,560	22,559,032	22,563,022
830	22,695,624	22,635,490	22,675,192	22,647,936	22,652,752	22,674,380	22,659,166	22,623,962	22,673,838	22,635,330
860	22,660,692	22,672,074	22,664,990	22,644,728	22,642,864	22,630,138	22,636,832	22,642,280	22,651,046	22,623,690

**Table 4:** Optimal cost (in Saudi Riyal SAR) of model (9) based on Algorithm 2 for K=50 to K=860 scenarios and R=11 to R=20 realizations.

Sample Size	11	12	13	14	15	16	17	18	19	20
50	22,249,392	22,192,802	21,970,916	22,047,142	22,212,648	22,184,694	22,106,002	22,099,104	22,304,932	22,052,730
80	22,575,492	22,647,942	22,549,028	22,533,152	22,581,404	22,595,868	22,466,710	22,660,108	22,701,058	22,702,358
110	23,125,668	23,153,764	23,102,100	23,041,618	23,126,458	23,153,412	23,124,164	23,118,946	23,091,818	23,140,872
140	23,067,442	22,967,508	23,022,206	23,044,090	23,052,884	23,100,226	23,114,012	23,074,332	22,923,810	23,128,412
170	22,733,392	22,819,432	22,817,980	22,862,874	22,871,170	22,848,170	22,732,296	22,834,966	22,856,576	22,906,492
200	22,992,666	22,915,620	23,019,108	22,922,658	22,913,238	22,977,670	22,962,866	22,933,226	22,992,542	22,964,562
230	22,954,086	22,904,750	22,942,966	22,934,224	22,867,416	22,883,290	22,934,408	22,895,392	22,973,656	22,924,338
260	22,999,396	22,935,130	22,990,118	23,061,400	22,996,090	22,914,978	23,010,328	22,958,194	22,983,276	22,993,130
290	22,587,052	22,511,014	22,570,306	22,627,148	22,603,150	22,613,320	22,619,410	22,615,694	22,617,304	22,634,472
320	22,664,048	22,695,692	22,639,600	22,707,522	22,716,420	22,753,308	22,713,382	22,669,380	22,723,230	22,734,278
350	22,633,188	22,611,582	22,608,832	22,654,540	22,598,510	22,602,920	22,597,876	22,602,526	22,597,946	22,635,430
380	22,620,336	22,675,614	22,650,702	22,621,408	22,645,424	22,625,120	22,692,752	22,659,200	22,643,590	22,675,280
410	22,762,290	22,774,240	22,737,504	22,713,468	22,758,334	22,739,174	22,781,474	22,753,202	22,727,940	22,754,310
440	22,621,666	22,619,300	22,641,762	22,590,016	22,636,126	22,617,230	22,634,554	22,597,860	22,659,590	22,601,596
470	22,615,676	22,596,638	22,571,348	22,580,078	22,531,980	22,573,886	22,581,134	22,570,440	22,586,898	22,584,838
500	22,640,810	22,617,506	22,631,338	22,694,764	22,672,802	22,711,946	22,699,262	22,664,782	22,656,392	22,707,886
530	22,532,612	22,523,770	22,496,352	22,527,724	22,504,102	22,550,588	22,530,438	22,521,800	22,473,192	22,530,806
560	22,653,770	22,689,972	22,697,422	22,716,862	22,671,716	22,705,676	22,637,370	22,682,956	22,689,030	22,692,382
590	22,624,788	22,647,334	22,615,382	22,666,100	22,628,862	22,627,646	22,654,536	22,665,720	22,662,434	22,654,534
620	22,539,118	22,561,548	22,562,272	22,569,628	22,592,958	22,560,592	22,573,762	22,552,834	22,553,254	22,562,266
650	22,508,556	22,537,404	22,520,592	22,539,984	22,545,440	22,561,712	22,514,016	22,505,132	22,548,260	22,560,968
680	22,559,640	22,578,104	22,580,518	22,604,592	22,623,030	22,551,958	22,536,640	22,552,938	22,577,580	22,591,406
710	22,745,774	22,719,346	22,698,272	22,729,826	22,744,698	22,758,930	22,750,384	22,700,350	22,748,522	22,722,210
740	22,701,916	22,737,038	22,713,906	22,724,550	22,684,052	22,712,630	22,711,882	22,699,240	22,709,156	22,725,202
770	22,695,032	22,722,628	22,698,262	22,641,372	22,718,944	22,671,872	22,693,706	22,646,232	22,712,832	22,662,506
800	22,543,436	22,580,192	22,582,996	22,543,788	22,604,186	2,257,077	22,575,264	22,577,728	22,603,708	22,558,152
830	22,633,486	22,634,216	22,660,650	22,650,060	22,656,632	22,667,046	22,654,776	22,634,866	22,653,400	22,676,644
860	22,648,034	22,662,028	22,664,396	22,678,954	22,667,786	22,624,580	22,637,904	22,686,834	22,638,014	22,668,170

**Table 5:** Optimal cost (in Saudi Riyal SAR) of model (9) based on Algorithm 2 for K=50 to K=860 scenarios and R=21 to R=30 realizations.

Sample Size	21	22	23	24	25	26	27	28	29	30
50	22,237,866	22,064,712	22,288,522	22,096,832	22,233,890	22,129,082	22,048,730	22,052,806	22,085,466	22,246,550
80	22,650,972	22,661,502	22,658,058	22,568,156	22,704,108	22,599,496	22,696,514	22,646,992	22,628,374	22,569,252
110	23,134,602	23,092,242	23,054,206	23,106,758	23,144,746	23,095,804	23,167,000	23,103,778	23,135,168	23,107,404
140	23,024,374	23,030,506	23,127,334	23,101,280	23,050,950	23,090,398	22,956,588	23,135,850	23,061,208	23,009,344
170	22,830,968	22,818,024	22,787,504	22,866,296	22,788,274	22,780,852	22,787,238	22,890,184	22,859,100	22,808,144
200	22,928,626	22,980,684	22,860,022	22,941,820	22,894,202	23,008,310	22,962,150	23,004,740	22,954,904	22,967,182
230	22,942,948	22,906,622	22,954,188	22,955,908	22,942,782	22,886,334	22,922,434	22,899,362	22,904,340	22,905,404
260	23,058,666	23,060,606	23,033,862	23,064,302	23,007,438	22,927,486	22,979,692	23,001,154	23,039,638	22,913,916
290	22,577,360	22,561,212	22,583,880	22,625,728	22,606,304	22,535,880	22,623,660	22,624,678	22,587,096	22,592,158
320	22,619,502	22,765,186	22,743,140	22,681,730	22,702,648	22,719,896	22,720,048	22,725,620	22,733,810	22,744,246
350	22,619,568	22,636,468	22,575,310	22,632,842	22,602,062	22,675,444	22,568,672	22,587,778	22,638,008	22,622,098
380	22,643,366	22,680,562	22,640,694	22,688,024	22,667,932	22,672,518	22,655,800	22,725,732	22,652,448	22,698,922
410	22,748,352	22,780,612	22,729,978	22,778,038	22,744,232	22,806,572	22,757,660	22,750,522	22,735,188	22,677,712
440	22,615,162	22,578,912	22,567,592	22,597,358	22,584,560	22,627,420	22,631,510	22,588,666	22,594,068	22,590,898
470	22,527,668	22,572,508	22,587,706	22,575,770	22,596,904	22,609,268	22,597,640	22,590,878	22,577,684	22,538,274
500	22,670,038	22,703,904	22,647,926	22,701,692	22,619,188	22,663,306	22,713,766	22,726,326	22,708,788	22,678,846
530	22,519,292	22,533,618	22,523,792	22,545,326	22,529,342	22,517,132	22,533,528	22,532,024	22,565,590	22,518,608
560	22,670,202	22,714,546	22,681,598	22,702,494	22,678,016	22,696,834	22,700,454	22,702,174	22,692,446	22,666,224
590	22,631,486	22,604,534	22,648,134	22,659,322	22,696,018	22,655,770	22,664,946	22,655,822	22,660,228	22,675,754
620	22,552,158	22,549,850	22,538,270	22,533,202	22,574,864	22,561,412	22,560,600	22,570,524	22,561,300	22,554,800
650	22,561,524	22,523,800	22,533,300	22,560,212	22,546,936	22,477,888	22,534,814	22,525,250	22,535,680	22,528,842
680	22,600,772	22,551,168	22,580,086	22,584,996	22,610,590	22,566,730	22,536,922	22,581,654	22,564,910	22,583,512
710	22,705,436	22,766,484	22,678,778	22,759,908	22,757,572	22,690,552	22,755,686	22,728,356	22,712,014	22,726,842
740	22,710,482	22,697,678	22,727,422	22,713,178	22,695,716	22,740,506	22,700,376	22,705,502	22,690,272	22,733,846
770	22,672,164	22,662,026	22,662,612	22,649,378	22,613,338	22,682,678	22,677,704	22,702,016	22,668,980	22,708,340
800	22,579,134	22,602,006	22,596,470	22,563,876	22,570,878	22,606,128	22,581,400	22,572,412	22,604,128	22,579,028
830	22,661,114	22,669,410	22,663,714	22,665,346	22,686,492	22,666,672	22,671,736	22,649,264	22,654,324	22,660,886
860	22,669,478	22,674,124	22,687,452	22,666,022	22,660,216	22,648,846	22,687,594	22,668,558	22,660,682	22,670,380

**Table 6:** Average optimal cost (in Saudi Riyal SAR) and variance of model (9) for sample sizes (number of scenarios) K=50 to K=860.

Sample Size	Average Optimal Cost	Variance	Standard Deviation
50	22,156,582	8,697,925,055	93,263
80	22,630,090	3,495,438,256	59,122
110	23,120,632	1,432,050,713	37,842
140	23,046,148	3,094,836,083	55,631
170	22,830,125	2,466,269,611	49,662
200	22,952,844	2,114,211,870	45,981
230	22,924,309	1,198,517,444	34,620
260	22,992,852	2,334,093,827	48,312
290	22,599,662	918,263,472	30,303
320	22,706,940	1,429,359,480	37,807
350	22,616,519	679,135,766	26,060
380	22,655,447	707,605,554	26,601
410	22,747,871	652,405,535	25,542
440	22,606,725	701,139,016	26,479
470	22,580,160	631,900,450	25,138
500	22,675,607	901,529,494	30,025
530	22,522,852	338,798,841	18,406
560	22,687,002	486,384,318	22,054
590	22,651,531	464,648,784	21,556
620	22,559,382	444,399,275	21,081
650	22,533,254	433,661,822	20,825
680	22,577,068	474,443,172	21,782
710	22,729,714	683,940,335	26,152
740	22,712,143	290,062,713	17,031
770	22,677,892	615,670,883	24,813
800	22,579,432	410,792,100	20,268
830	22,658,147	281,864,861	16,789
860	22,657,980	326,299,652	18,064

**Table 7:** Statistical analysis of average optimal cost (in Saudi Riyal SAR) and standard deviation of model (9) across varying sample sizes (number of scenarios) K=50 to K=860.

# Scenarios	Average	Standard Deviation	T-Statistic	F-Statistic
50	22,156,582	93,263		
80	22,630,090	59,122	23.414	0.382
110	23,120,632	37,842	39.218	0.383
140	23,046,148	55,631	−6.222	2.559
170	22,830,125	49,662	−15.467	0.785
200	22,952,844	45,981	9.841	0.857
230	22,924,309	34,620	−2.766	0.574
260	22,992,852	48,312	6.071	1.813
290	22,599,662	30,303	−37.490	0.411
320	22,706,940	37,807	11.886	1.604
350	22,616,519	26,060	−10.700	0.439
380	22,655,447	26,601	6.092	1.062
410	22,747,871	25,542	13.691	0.916
440	22,606,725	26,479	−20.793	1.133
470	22,580,160	25,138	−3.948	0.914
500	22,675,607	30,025	12.863	1.406
530	22,522,852	18,406	−23.007	0.380
560	22,687,002	22,054	30.692	1.345
590	22,651,531	21,556	−6.124	1.005
620	22,559,382	21,081	−16.248	0.964
650	22,533,254	20,825	−4.658	0.980
680	22,577,068	21,782	7.690	1.036
710	22,729,714	26,152	24.163	1.529
740	22,712,143	17,031	−3.060	0.416
770	22,677,892	24,813	−5.901	1.996
800	22,579,432	20,268	−17.057	0.643
830	22,658,147	16,789	16.202	0.765
860	22,657,980	18,064	−0.255	1.111

**Table 8:** Upper and lower bounds for Stage 1 solution for R=30 realizations.

Realization	Upper Bound	Lower Bound	Gap %
1	22,954,892	22,652,406	1.318
2	22,924,610	22,652,406	1.187
3	22,956,590	22,652,406	1.325
4	22,975,284	22,652,406	1.405
5	22,969,024	22,652,406	1.378
6	22,947,760	22,652,406	1.287
7	22,963,016	22,652,406	1.353
8	22,932,946	22,652,406	1.223
9	22,968,390	22,652,406	1.376
10	22,952,404	22,652,406	1.307
11	22,928,422	22,652,406	1.204
12	22,989,526	22,652,406	1.466
13	22,960,652	22,652,406	1.342
14	22,973,002	22,652,406	1.396
15	22,974,328	22,652,406	1.401
16	22,992,136	22,652,406	1.478
17	22,914,500	22,652,406	1.144
18	22,938,440	22,652,406	1.247
19	22,926,550	22,652,406	1.196
20	22,929,458	22,652,406	1.208
21	22,948,440	22,652,406	1.290
22	22,982,712	22,652,406	1.437
23	22,951,656	22,652,406	1.304
24	22,962,418	22,652,406	1.350
25	22,959,582	22,652,406	1.338
26	22,947,288	22,652,406	1.285
27	22,959,474	22,652,406	1.337
28	22,950,028	22,652,406	1.297
29	22,957,338	22,652,406	1.328
30	22,983,926	22,652,406	1.442

**Table 9:** Stage 1 solution for R=30 realizations of the optimal number of scenarios at $K^{*}=860$.

Realization	SABIC 1	SABIC 2	SABIC 3	SIPCHEM	*Z*
1	0	0	705	3959	0
2	0	0	630	3962	0
3	0	0	311	3993	0
4	0	0	212	4000	0
5	0	0	390	3985	0
6	0	0	70	4014	0
7	0	0	186	4002	0
8	0	0	586	3971	0
9	0	0	935	3931	0
10	0	0	463	3977	0
11	0	0	727	3951	0
12	0	0	769	3954	0
13	0	0	528	3976	0
14	0	0	599	3969	0
15	0	0	980	3938	0
16	0	0	395	3985	0
17	0	0	478	3979	0
18	0	0	977	3939	0
19	0	0	474	3976	0
20	0	0	728	3959	0
21	0	0	935	3931	0
22	0	0	575	3972	0
23	0	0	977	3939	0
24	0	0	490	3977	0
25	0	0	599	3969	0
26	0	0	515	3975	0
27	0	0	910	3946	0
28	0	0	635	3964	0
29	0	0	493	3977	0
30	0	0	601	3967	0

**Table 10:** Investment plan for Stage 1 based on two-stage bound portfolio optimization model (9).

Period	Interest Rate	Liabilities (in SR)	Cash Accumulated at the Beginning of Period *t* (in SR)	Cash Flow (in SR)	Total Interest (in SR)	Total Income (in SR)	Cash Accumulated at the End of Period *t* (in SR)
1	0.0148	1,009,895	0	5,006,907	0	5,006,907	3,997,012
2	0.0317	2,275,167	3,997,012	5,006,907	4,123,717	9,130,624	6,855,457
3	0.0112	3,064,511	6,855,457	5,006,907	6,932,238	1,1939,145	8,874,634
4	0.0112	4,086,015	8,874,634	5,006,907	8,974,030	1,3980,937	9,894,922

**Table 11:** Computational cost of Algorithm 2 based on different number of scenarios.

Number of Scenarios	20	40	60	80	90	100	500	1000	2000	5000
Computational Cost (in seconds)	0.02	0.024	0.067	0.207	0.157	0.036	0.1	1.207	64.94	3.94

## Data Availability

Saudi Sukuk data presented in this study can be obtained from https://www.tadawulgroup.sa/wps/portal/ accessed on 23 October 2025.

## References

[1] Ardakani O. (2024). Portfolio optimization with transfer entropy constraints. Int. Rev. Financ. Anal..

[2] Yan X., Yang H., Yu Z., Zhang S., Zheng X. (2023). Portfolio optimization: A return-on-equity network analysis. IEEE Trans. Comput. Soc. Syst..

[3] Novais R., Wanke P., Antunes J., Tan Y. (2022). Portfolio optimization with a mean-entropy-mutual information model. Entropy.

[4] Markowitz H. (1952). Portfolio Selection. J. Financ..

[5] Shapiro J.F. (1988). Stochastic programming models for dedicated portfolio selection. Mathematical Models for Decision Support.

[6] Bradley S.P., Crane D.B. (1972). A dynamic model for bond portfolio management. Manag. Sci..

[7] Hu Y., Chen X., He N. (2020). Sample Complexity of Sample Average Approximation for Conditional Stochastic Optimization. SIAM J. Optim..

[8] Jiang J., Li S. (2021). On complexity of multistage stochastic programs under heavy tailed distributions. Oper. Res. Lett..

[9] Bertsimas D., Gupta V., Kallus N. (2018). Robust Sample Average Approximation. Math. Program..

[10] Mercurio P.J., Wu Y., Xie H. (2020). An Entropy-Based Approach to Portfolio Optimization. Entropy.

[11] Prékopa A. (1995). Stochastic Programming.

[12] Shapiro A. Monte Carlo simulation approach to stochastic programming. Proceedings of the 2001 Winter Simulation Conference (Cat. No. 01CH37304).

[13] Brandimarte P. (2014). Handbook in Monte Carlo Simulation: Applications in Financial Engineering, Risk Management, and Economics.

[14] Alreshidi N.A., Mrad M., Subasi E., Subasi M.M. (2020). Two-stage bond portfolio optimization and its application to Saudi Sukuk Market. Ann. Oper. Res..

[15] Alkailany M.A., Abdalrazzaq M.S. (2022). A new bond portfolio optimization model as two-stage stochastic programming problems in US market. Int. J. Nonlinear Anal. Appl..

[16] Hodges S.D., Schaefer S.M. (1977). A model for bond portfolio improvement. J. Financ. Quant. Anal..

[17] Ronn E.I. (1987). A new linear programming approach to bond portfolio management. J. Financ. Quant. Anal..

[18] Korn O., Koziol C. (2006). Bond portfolio optimization: A risk-return approach. J. Fixed Income.

[19] Stoyan S.J., Kwon R.H. (2011). A stochastic-goal mixed-integer programming approach for integrated stock and bond portfolio optimization. Comput. Ind. Eng..

[20] He F., Qu R. (2014). A two-stage stochastic mixed-integer program modelling and hybrid solution approach to portfolio selection problems. Inf. Sci..

[21] Maggioni F., Allevi E., Tomasgard A. (2020). Bounds in multi-horizon stochastic programs. Ann. Oper. Res..

[22] Salo A., Stoyan S., Wallace S., Zenios S. (2024). Fifty Years of Portfolio Optimization: A Retrospective and Prospects. Eur. J. Oper. Res..

[23] Zhang W., Wang K., Jacquillat A., Wang S. (2023). Optimized scenario reduction: Solving large-scale stochastic programs with quality guarantees. INFORMS J. Comput..

[24] Henrion R., Römisch W. (2022). Problem-based optimal scenario generation and reduction in stochastic programming. Math. Program..

[25] Chan T., Lin B., Saxe S. (2025). Machine Learning–Augmented Optimization of Large Bilevel and Two-Stage Stochastic Programs: Application to Cycling Network Design. Manuf. Serv. Oper. Manag..

[26] Dantzig G., Infanger G. (1993). Multi-stage stochastic linear programs for portfolio optimization. Ann. Oper. Res..

[27] Mulvey J., Vladimirou H. (1992). Stochastic network programming for financial planning problems. Manag. Sci..

[28] Zenios S. (1995). Asset/liability management under uncertainty for fixed-income securities. Ann. Oper. Res..

[29] Cariño D.R., Kent T., Myers D.H., Stacy C., Sylvanus M., Turner A.L., Watanabe K., Ziemba W.T. (1994). The Russell-Yasuda Kasai model: An asset/liability model for a Japanese insurance company using multistage stochastic programming. Interfaces.

[30] Dentcheva D., Ruszczynski A. (2006). Portfolio optimization with stochastic dominance constraints. J. Bank. Financ..

[31] Gülpınar N., Rustem B., Settergren R. (2016). Robust portfolio optimization under downside risk measures. Comput. Manag. Sci..

[32] Shapiro A., Ugurlu K. (2016). Decomposability and time consistency of risk averse multistage programs. Oper. Res. Lett..

[33] Jobst N., Mitra G., Zenios S. (2009). Integrating market and credit risk: A simulation and optimisation perspective. J. Bank. Financ..

[34] Fábián C., Mitra G., Roman D. (2011). Processing second-order stochastic dominance models using cutting-plane representations. Math. Program..

[35] Bertsimas D., Kallus N. (2020). From predictive to prescriptive analytics. Manag. Sci..

[36] Consigli G., Dentcheva D., Maggioni F., Micheli A. (2025). Asset Liability Management under Sequential Stochastic Dominance Constraints. arXiv.

[37] Yang R., Yu H., Li Z., Zhao X. (2022). A Multistage Stochastic Programming Model with Multiple Objectives for Optimal Issuance of Corporate Bonds. Discret. Dyn. Nat. Soc..

[38] Basciftci B., Ahmed S., Duenyas I., Shen Z. (2024). Adaptive Two-Stage Stochastic Programming with Applications to Capacity Expansion Planning. Manuf. Serv. Oper. Manag..

[39] Rubinstein R.Y., Shapiro A. (1993). Discrete Event Systems: Sensitivity Analysis and Stochastic Optimization by the Score Function Method.

[40] Geyer C.J., Thompson E.A. (1992). Constrained Monte Carlo maximum likelihood for dependent data. J. R. Stat. Soc. Ser. B (Methodol.).

[41] Infanger G. (1992). Planning Under Uncertainty Solving Large-Scale Stochastic Linear Programs.

[42] Plambeck E.L., Fu B.R., Robinson S.M., Suri R. (1996). Sample-path optimization of convex stochastic performance functions. Math. Program..

[43] Gurkan G., Ozge A.Y., Robinson T. Sample-path optimization in simulation. Proceedings of the Winter Simulation Conference.

[44] Shapiro A., Wardi Y. (1996). Convergence analysis of gradient descent stochastic algorithms. J. Optim. Theory Appl..

[45] Shapiro A., Homem-de Mello T. (1998). A simulation-based approach to two-stage stochastic programming with recourse. Math. Program..

[46] Verweij B., Ahmed S., Kleywegt A.J., Nemhauser G., Shapiro A. (2003). The sample average approximation method applied to stochastic routing problems: A computational study. Comput. Optim. Appl..

[47] Royset J., Polak E. (2004). Reliability-based optimal design using sample average approximations. Probabilistic Eng. Mech..

[48] Santoso T., Ahmed S., Goetschalckx M., Shapiro A. (2005). A stochastic programming approach for supply chain network design under uncertainty. Eur. J. Oper. Res..

[49] Jirutitijaroen P., Singh C. (2008). Reliability constrained multi-area adequacy planning using stochastic programming with sample-average approximations. IEEE Trans. Power Syst..

[50] Keller B., Bayraksan G. (2009). Scheduling jobs sharing multiple resources under uncertainty: A stochastic programming approach. IIE Trans..

[51] Hilli P., Koivu M., Pennanen T., Ranne A. (2007). A stochastic programming model for asset liability management of a Finnish pension company. Ann. Oper. Res..

[52] Homem-de Mello T., Bayraksan G. (2014). Monte Carlo sampling-based methods for stochastic optimization. Surv. Oper. Res. Manag. Sci..

[53] Pasupathy R., Song Y. (2021). Adaptive Sequential Sample Average Approximation for Two-Stage Stochastic Linear Programs. SIAM J. Optim..

[54] Kannan R., Bayraksan G., Luedtke J. (2025). Data-Driven Sample Average Approximation with Covariate Information. Oper. Res..

[55] Lew T., Sahin A., Chen X., Harchaoui Z. (2022). Sample Average Approximation for Stochastic Programs with Equality Constraints: Complexity and Algorithms. arXiv.

[56] Alnaggar A., Gzara F., Bookbinder J.H. (2020). Distribution planning with random demand and recourse in a transshipment network. EURO J. Transp. Logist..

[57] Afshar T.A. (2013). Compare and contrast Sukuk (Islamic Bonds) with conventional bonds, are they compatible?. J. Glob. Bus. Manag..

[58] Alswaidan M.W. (2013). Saudi Arabian Sukuk Market: Recent Trends and Development. Bus. Rev. Camb..

